# Low-cost, rapid and reliable DNA extraction from mammalian organoids using a *C. elegans* toolbox

**DOI:** 10.17912/micropub.biology.001240

**Published:** 2024-06-24

**Authors:** Jonas Mars, Gabriella S. Darmasaputra, Matilde Galli, Hendrik C. Korswagen

**Affiliations:** 1 Hubrecht Institute, Royal Netherlands Academy of Arts and Sciences and University Medical Center Utrecht, The Netherlands; 2 Department of Biology, Institute of Biodynamics and Biocomplexity, Developmental Biology, Utrecht University, 3584 CH, Utrecht, The Netherlands

## Abstract

High-quality DNA extraction from organoids is an important step in molecular genetics research. Here, we show that a lysis buffer from the field of *Caenorhabditis elegans* research, called Single Worm Lysis Buffer (SWLB), is a low-cost, yet reliable method for DNA extraction from mammalian organoids. SWLB is superior in terms of price, storage, hands-on time and sustainability compared to current standardized DNA extraction protocols, while equally effective. This work indicates that it is useful to compare methods from different model systems, such as mammalian organoids and invertebrate nematodes, to find useful alternatives for research methodologies.

**Figure 1. Single Worm Lysis Buffer can be used to reliably extract DNA from mammalian organoids using a low-cost and rapid protocol:**
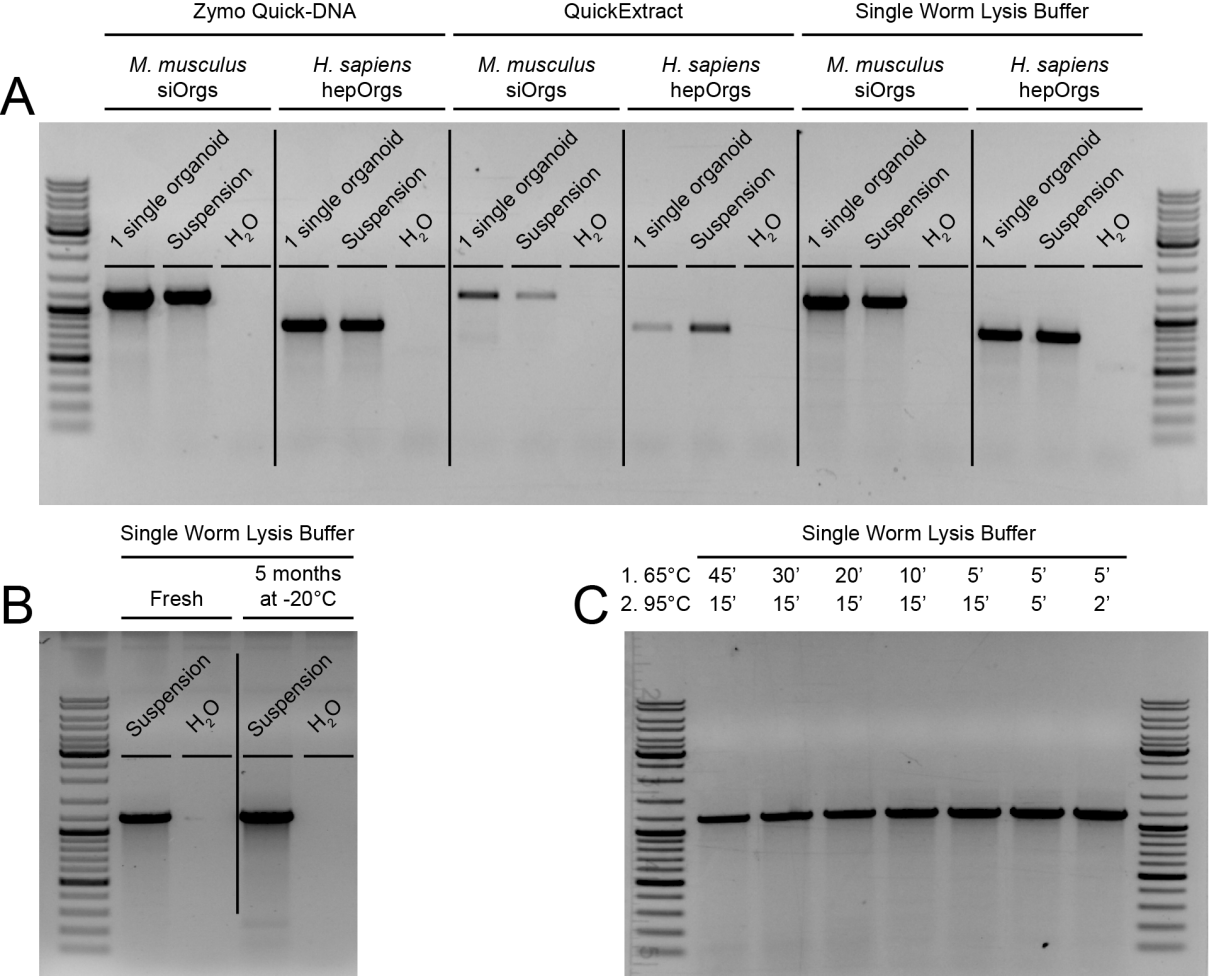
(A) Agarose gel electrophoresis of PCR products obtained using DNA extracted from two organoid lines using one of three indicated methods: Zymo Quick-DNA microprep kit, Biosearch Technologies' QuickExtract Solution or Single Worm Lysis Buffer. For mouse small intestinal organoids ( *M. musculus* siOrgs), *Eva1c* PCR products are visible at the expected size of 1162 bp; for human fetal hepatocyte organoids ( *H. sapiens* hepOrgs), *E2F8* PCR products are visible at the expected size of 790 bp. For both organoid lines, either single organoids or 1 µl of organoid suspension was used and water controls were included for all experiments. (B) Agarose gel electrophoresis showing that 5-month storage at -20°C of a lysed *M. musculus* small intestinal organoid sample using Single Worm Lysis Buffer does not affect the stability of the extracted DNA. 1 µl of organoid suspension was used for DNA extraction; *Eva1c* PCR products are visible at the expected size of 1162 bp. (C) Agarose gel electrophoresis comparing different incubation times at 65°C and 95°C of the Single Worm Lysis Buffer protocol for *M. musculus* small intestinal organoid samples. 5 minutes at 65°C, followed by 2 minutes at 95°C is sufficient to prepare organoid lysate that will result in prominent, single bands after PCR. 1 µl of organoid suspension was used for DNA extraction; *Eva1c* PCR products are visible at the expected size of 1162 bp for all tested incubation times. All experiments were performed at least three times, the images show representative results.

## Description

DNA extraction is a key process in molecular genetics to obtain material for genotyping or cloning experiments. Organoids are three-dimensional cellular structures derived from animal stem cells that resemble the complexity and dynamics of a developing organ. They are widely applied in the field of molecular genetics to study processes related to development or regenerative medicine *in vitro* . To extract DNA from a biological sample such as an organoid, different methods are used. For example, DNA extraction kits such as the Zymo Quick-DNA microprep kit (Zymo, #D3021) supply the experimenter with reagents and materials to obtain DNA from cells or tissue using a silica-membrane based spin column technique. Biosearch Technologies provides a simpler DNA extraction protocol using QuickExtract Solution (Biosearch Technologies, #QE09050), where a single lysis buffer can be mixed with the sample to retrieve DNA via vortexing and heating steps. Both methods only require ca. 15 minutes of hands-on time to obtain DNA, but they are relatively expensive to use with prices ranging from €2 (Zymo Quick-DNA) to €7 (QuickExtract) per reaction. Although this could be a reasonable price for a few samples, it can get expensive when handling many samples, for example when screening clones.

In *C. elegans* research, DNA can be extracted from single animals after resuspending them in an in-house made lysis buffer named Single Worm Lysis Buffer (SWLB), supplemented with proteinase K (Williams et al., 1992) . Then, a thermocycler-based heating protocol of 45 minutes at 65°C, followed by 15 minutes at 95°C is used to extract the DNA from the animal. One of the advantages of SWLB is that its lysis buffer can be made in-house at a very low price (€0.03 per reaction), which contrasts more expensive supplier kits, making the SWLB method the golden standard for DNA extraction from *C. elegans* .

Despite its potential as a cheap, yet highly reliable DNA extraction method, SWLB has not been widely applied outside the field of *C. elegans* to date. Here, we set out to investigate whether DNA can be reliably extracted from mammalian organoids using a *C. elegans* -based SWLB protocol. We compared three different methodologies for DNA extraction: the Zymo Quick-DNA microprep kit, QuickExtract Solution and Single Worm Lysis Buffer. We used two types of mammalian organoids: mouse small intestinal organoids (siOrgs) and human fetal hepatocyte organoids (hepOrgs). Furthermore, we tested if we could extract DNA from organoids in suspension (with an estimated density of 200 cells/µl) as well as from individual organoids (approximately 100 cells), as the latter is a prerequisite for genotyping single clonal organoids after a selection. We used PCR to amplify the DNA for 35 cycles in all cases and gel electrophoresis to visualize our amplified products. We obtained prominent bands with little background for all three methods ( Figure 1A ). Of note, QuickExtract performed less well in our hands, whereas the Zymo Quick-DNA and SWLB methods gave similar results. We conclude that SWLB can be used to extract DNA from mammalian organoids with high reliability. Importantly, storage of a mouse siOrgs suspension sample, after lysis using SWLB, for at least five months at -20°C did not affect this result ( Figure 1B ), showing that DNA extracted using SWLB is stable over longer time periods.

Finally, we hypothesized that the 1-hour incubation step of the SWLB protocol, necessary to break down the tough cuticle of *C. elegans* , could be shortened for organoids. To test the minimal lysis time needed for DNA extraction from mouse siOrgs using SWLB, we incubated resuspended mouse siOrgs for different times in the thermocycler. We were able to successfully extract DNA from mouse siOrgs, again shown by prominent bands without any background, using a thermocycler lysis protocol of only 5 minutes at 65°C, followed by 2 minutes at 95°C ( Figure 1C ).

Thus, organoid DNA extraction using Single Worm Lysis Buffer is as reliable as commercial DNA extraction protocols, such as the Zymo Quick-DNA microprep kit or Biosearch Technologies' QuickExtract kit. However, the costs and hands-on time are much lower for the SWLB protocol, as SWLB can be made in-house at a fraction of the price. Moreover, Zymo Quick-DNA and QuickExtract protocols require hands-on time for column purification or vortexing, respectively, whereas the SWLB protocol only requires a 7-minute incubation in a thermocycler. Finally, the SWLB protocol does not require single-use plastic spin columns, making it a more sustainable choice for researchers looking for DNA extraction method alternatives.

In summary, we present a reliable and cost-effective method for DNA extraction from mammalian organoids. The effectiveness of a *C. elegans* lysis buffer on organoid DNA extraction shows the value of comparing methodologies from different model systems to enhance research effectiveness. Single Worm Lysis Buffer is cheap, as it can be made in-house using standard chemicals, can be stored at room temperature, requires little hands-on time and is a sustainable choice when considering plastic waste from spin columns. We expect that the SWLB protocol can be widely applied for DNA extraction from various model systems, simplifying this key step in molecular genetics research.

## Methods

Organoid culturing

Rosa26 ^CreERT2^ mouse small intestinal organoids were cultured as described before (Gehart et al., 2019) . Human fetal hepatocyte organoids were cultured as described before (Hendriks et al., 2021) .

DNA extraction

DNA extraction was performed using 1 µl of organoid suspension (approximately 200 cells/µl), 1 single picked organoid (200 µm in diameter with approximately 100 cells) or 1 µl of MilliQ water, according to the manufacturer's instructions (Zymo Quick-DNA microprep kit or QuickExtract) or using the Single Worm Lysis Buffer protocol. For the Zymo Quick-DNA microprep kit, the manufacturers protocol for Cell Monolayer Samples was followed and DNA was eluted using 15 µl of DNA Elution Buffer. Eluted DNA was stored at -20°C and used within one week. For QuickExtract, organoid samples were added to 500 µl of QuickExtract Solution, followed by the vortexing and heating steps as indicating in the manufacturer's protocol. DNA was stored at -20°C and used within one week.

Single Worm Lysis Buffer was made using MilliQ water supplemented with 50 mM KCl, 2.5 mM MgCl _2_ , 10 mM Tris HCl (pH = 8.3), 0.45% of NP40 and 0.45% Tween-20. Proteinase K was added to Single Worm Lysis Buffer (1:20) to a final concentration of 1 mg/ml and the mixture was briefly vortexed before addition of organoid samples. Of note, in contrast to other described methods for SWLB-based DNA extraction, we find that it is not necessary to freeze the samples before incubation. Instead, we directly proceed to the heating steps, performed in a thermocycler (Biorad #1861096).

PCR conditions and gel electrophoresis

For Zymo Quick-DNA eluate and Single Worm Lysis Buffer lysate, 1 µl of lysate was used for PCR amplification. For QuickExtract, 5 µl of lysate was used. PCR was performed in a thermocycler (Biorad #1861096) using a 20 µl PCR reaction consisting of the following mastermix:

**Table:** 

**Reagent**	**Volume**
DNA lysate	Depending on extraction method
5X GoTaq Green Reaction Buffer (Promega)	4 µl
MgCl _2_ (25 mM)	1.6 µl
Forward primer (10 µM)	0.5 µl
Reverse primer (10 µM)	0.5 µl
dNTPs (10 µM)	0.5 µl
Taq DNA polymerase (5U/µl)	0.5 µl
MilliQ water	To 20 µl

Primers for mouse small intestinal organoids are GGAATGTCCTTCATAAGGGC (forward) and CCTGCTTTCTCTACACTCCC (reverse), using an annealing temperature of 58°C and 60 seconds extension time. Primers for human fetal hepatocyte organoids are TCTGCGCCCAGTAGCCCTATCA (forward) and ATGAGGGACGGTGCTGTCAGCT (reverse), using an annealing temperature of 60°C and 30 seconds extension time. Otherwise, PCR conditions were as follows:

**Table:** 

**Step**	**Temperature**	**Duration**	
Initial denaturation	94°C	5 minutes	
Denaturation	94°C	30 seconds	35 cycles
Annealing	Depending on primer set	30 seconds	
Extension	72°C	Depending on primer set	
Final extension	72°C	5 minutes	

After PCR amplification, 5 µl of PCR product was loaded on a 1% TAE agarose gel with Ethidium Bromide (1:10.000) and visualized using the Biorad Molecular Imager Gel Doc XR+.

## Reagents

**Table:** 

**Organoid line**	**Supplier**	**Reference**
Rosa26 ^CreERT2^	Gift from the Clevers lab, Hubrecht Institute	Previously used in (Souilhol et al., 2016) .
Human fetal hepatocyte organoids	Gift from Sarina Shabso (Organoid group, Hubrecht Institute)	Established in (Hendriks et al., 2021) .
		
**Reagent**	**Supplier**	**Catalog number**
Quick-DNA microprep kit	Zymo	D3021
QuickExtract Solution	Biosearch Technologies	QE09050
KCl	Sigma Aldrich	7447-40-7
MgCl _2_	Promega	A3513
UltraPure Tris	ThermoFisher Scientific	15504020
NP40	Sigma Aldrich	74385
Tween-20	Sigma Aldrich	P1379
Proteinase K	Promega	A5051
Gotaq Green Reaction Buffer	Promega	M7911
Primers	Integrated DNA Technologies	-
Deoxynucleotide (dNTP) Solution Mix	Promega	U1410
Taq polymerase	Made in-house	-
Ethidium Bromide	Sigma	E1510
MilliQ		
